# The Critical Role of Small RNAs in Regulating Plant Innate Immunity

**DOI:** 10.3390/biom11020184

**Published:** 2021-01-29

**Authors:** Saquib Waheed, Muhammad Anwar, Muhammad Asif Saleem, Jinsong Wu, Muhammad Tayyab, Zhangli Hu

**Affiliations:** 1 College of Horticulture, Fujian Agriculture and Forestry University, Fuzhou 350002, China; swaheed022@gmail.com; 2 Guangdong Technology Research Center for Marine Algal Bioengineering, Guangdong Key Laboratory of Plant Epigenetics, College of Life Sciences and Oceanography, Shenzhen University, Shenzhen 518060, China; 3 Key Laboratory of Optoelectronic Devices and Systems of Ministry of Education and Guangdong Province, College of Optoelectronic Engineering, Shenzhen University, Shenzhen 518060, China; 4 Department of Plant Breeding and Genetics, Bahauddin Zakariya University, Multan 60800, Pakistan; drasifsaleem@bzu.edu.pk; 5 Shenzhen Key Laboratory of Marine Bioresource & Eco-Environmental Science, Longhua Innovation Institute for Biotechnology, Shenzhen University, Shenzhen 518060, China; 1800252001@email.szu.edu.cn; 6 Key Laboratory of Sugarcane Biology and Genetic Breeding, Ministry of Agriculture and Forestry University, Fuzhou 350002, China; gulanwar85@gmail.com

**Keywords:** innate immunity, small RNAs, pathogens, PTI, ETI

## Abstract

Plants, due to their sessile nature, have an innate immune system that helps them to defend against different pathogen infections. The defense response of plants is composed of a highly regulated and complex molecular network, involving the extensive reprogramming of gene expression during the presence of pathogenic molecular signatures. Plants attain proper defense against pathogens through the transcriptional regulation of genes encoding defense regulatory proteins and hormone signaling pathways. Small RNAs are emerging as versatile regulators of plant development and act in different tiers of plant immunity, including pathogen-triggered immunity (PTI) and effector-triggered immunity (ETI). The versatile regulatory functions of small RNAs in plant growth and development and response to biotic and abiotic stresses have been widely studied in recent years. However, available information regarding the contribution of small RNAs in plant immunity against pathogens is more limited. This review article will focus on the role of small RNAs in innate immunity in plants.

## 1. Introduction

Plants are capable of developing a defense response against potentially pathogenic organisms through innate immunity. In general, the first level of defense in plants is based on the pre-existing physical barrier (cell wall) that hinders the penetration of the pathogens, as well as the accumulation of compounds that can be toxic to plants. This barrier is constitutive and may not be sufficient to stop the invasion of pathogens. Thus, plants also have to employ active defense mechanisms that are induced by the presence of pathogens. Studies related to model plants have shown that the plant immune system consists of a two-tier structure that perceives and responds in the presence of intracellular and extracellular pathogen signatures. Within the first tier, plants recognize the existence of pathogens through pathogen-associated molecular patterns (PAMPs) through plant receptors located on the cell surface, known as plant pattern recognition receptors (PRRs) [1]. This recognition triggers a basal defense response known as pattern-triggered immunity (PTI), which induces the production of reactive oxygen species (ROS) and nitric oxide (NO), the accumulation of salicylic acid (SA), the deposition of callose, and the expression of pathogenesis-related (PR) genes, to ultimately prevent non-adapted microbes from infecting the plant [2,3,4]. However, plants continuously produce ROS as a byproduct of metabolic processes, which induce an extracellular oxidative burst as part of the defense response [5]. ROS molecules have a double function, being able to act as an antimicrobial agent and also as an activator of defense responses through signaling such as superoxide radicals (O_2_-) and hydrogen peroxide (H_2_O_2_). The accumulation of H_2_O_2_ also favors the polymerization of lignin precursors in the cell wall and hinders the penetration of the pathogen. However, an excess of ROS molecules can be toxic to the plant itself, given its ability to irreversibly modify lipids, proteins, and nucleic acids, with consequent cell damage. Therefore, plants have mechanisms to maintain adequate levels of ROS in the cell to prevent oxidative damage through the production of compounds with antioxidant capacity (glutathione, ascorbate, sugars, flavonoids, alkaloids, carotenoids, or tocopherols) or enzymatic activities (glutathione S-transferases, superoxide dismutases, catalases, or peroxidases). Other than ROS, the excessive production of nitric oxide can trigger a response toward pathogen infection. Nitric oxide participates in different defense-associated processes by interacting with transduction pathways through involving kinase proteins and signaling hormonal pathways such as salicylic acid and jasmonic acid, and also functions in cell death programs [6]. The activation of the complex network and interactions between different signaling pathways lead to the induction of genes encoding pathogenesis-related (PR) proteins. PR proteins are classified into diverse families based on their sequence and functional homology [7]. Studies have shown that PR proteins have antimicrobial activity, including chitinases, β-1,3-glucanases, and thionins. During a pathogen attack, callose deposition and the accumulation of hydroxyproline-rich proteins in the cell wall are common plant responses that hinder the penetration of pathogens into plant tissues [8].

Certain pathogens, in turn, have developed mechanisms to overcome this first level of defense with the production of compounds capable of interfering with the PTI response, known as effector proteins. Likewise, plants recognize and counteract these effector proteins by using a second tier of defense through the production of resistance proteins (R proteins). This recognition is host specific and triggers a response called effector-triggered immunity (ETI) that is highly effective in counteracting the pathogen attack [9]. The ETI response is often associated with the hypersensitive response (HR), in which the controlled death of cells takes place at the infection site, limiting the spread of infection [10].

In an infection situation, plants also activate the production of secondary metabolites, such as phytoalexins, camalexin, and flavonoids, which can potentially participate in the defense against pathogens [11,12]. The recognition of pathogens also triggers a series of other responses in plants, such as depolarization of the plasma membrane and alterations in the flow of ions (entry of H^+^ and Ca^2+^; the release of K^+^ and Cl^−^). The entry of Ca^2+^ (second messenger in many cellular processes) activates signaling pathways through the participation of calcium-dependent protein kinases (CDPKs) [13]. CDPKs have emerged as important Ca^2+^ sensor proteins in transducing differential Ca^2+^ signatures, and overlapping CDPKs phosphorylate distinct substrates to regulate diverse plant immune responses, including the transcriptional reprogramming of immune genes. Transcription factors belonging to different families regulate the expression of defense genes during pathogen attack [14]. Among them are WRKY transcription factors, which recognize W type boxes (W-box) [15], AP2/ERF transcription factors (mainly associated with the signaling pathway of ethylene) [16], bZIP transcription factors (basic leucine zipper domain), and MYB transcription factors [17].

Hormones play a crucial role in disease resistance by regulating the defense mechanisms of plants [18,19]. Historically, salicylic acid has been associated with plant defense against biotrophic pathogens, while jasmonic acid and ethylene are associated with protection against necrotrophic pathogens [20]. The salicylic acid (SA) and jasmonic acid (JA)/ethylene (ET) routes are mutually antagonistic. However, synergistic interactions between these pathways have also been described, which suggests that the signaling network used by plants is dependent on both the lifestyle of the pathogen in the host plant and the host plant itself and also the tissue type or developmental stage [21]. Auxins, brassinosteroids, and gibberellic acid also play a fundamental role in the defense response of plants [22]. In *Arabidopsis*, abscisic acid (ABA) contributes to innate immunity during pathogen infection [23]. Plants also counter pathogen attack by systemic acquired resistance (SAR) [24] and induced systemic resistance (ISR) [25] through hormone signaling. SAR is induced after initial infection by a pathogen, manifests itself at sites distant from the area of infection, and is useful in counteracting the infection not only by the pathogen that initially triggered it but also by other pathogens [26]. The SAR response can be maintained for more extended periods (up to weeks) and is accompanied by an increase in the accumulation of SA that induces the expression of defense genes, such as PR genes. ISR is induced by soil microorganisms that colonize plant roots [27]. Like SAR, ISR is a systemic resistance process that depends on signaling induced by hormones, such as ethylene (ET) and jasmonic acid (JA). ISR is accompanied by the activation of pathogenesis-related genes. Treatment with specific chemical agents (beta-aminobutyric acid and benzothiadiazole) can also activate defense mechanisms typically associated with induced resistance [28].

**Figure 1:**
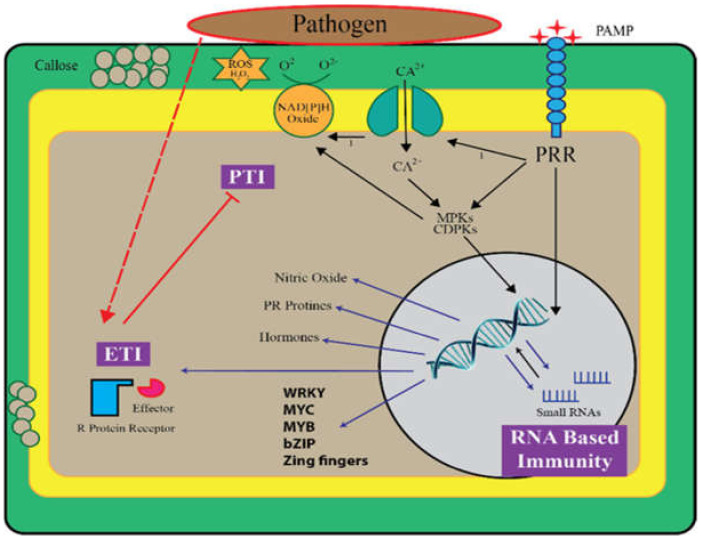
A mechanism for protecting plants against fungal and bacterial pathogens. Typical responses to basal pathogen-triggered immunity and pathogen-specific effector-triggered immunity and the RNA-based immune system are presented. PTI, pattern-triggered immunity; ETI, effector-triggered immunity; PR, pathogenesis-related; PRR, pattern recognition receptor; PAMP, pathogen-activated molecular pattern; CDPKs, calcium-dependent protein kinases; ROS, reactive oxygen species; MPKs, mitogen-activated protein kinases.

For a long time, it was considered that the PTI and ETI responses against plant pathogens were based on the transcriptional regulation of genes coding for proteins (defense proteins) and that these mechanisms were independent of RNA silencing (Figure 1). Recent studies have revealed that small RNAs are critical regulators of the plant immune response and are also an effective approach for crop improvement [29,30]. The defense response of plants against pathogens involves the mechanism of post-transcriptional gene regulation, which is mediated through the activity of small RNAs (sRNAs) [31,32]. Plants possess two main types of small non-coding endogenous RNAs, microRNAs (miRNAs) and small interference RNAs (siRNAs). These two classes of small RNAs are similar in size and range between 18–30 nt in length but differ in the biogenesis pathway, precursor structures, and modes of action [33]. Many sRNAs have been identified using high-throughput sequencing and bioinformatics tools, which are involved in sequence-specific gene regulation by small non-coding RNAs [34].

In plant biology, the scientific literature in recent years has increased dramatically regarding the crucial role of sRNAs in plant defenses. In this respect, several researchers have contributed some excellent reports on the role of sRNAs in plant defense mechanisms [31,35]. However, we think that a critical and comprehensive review article is required to elaborate the role of sRNAs in plant innate immunity. This review will therefore include a detailed discussion on current knowledge and recent progress regarding the role of sRNAs and their involvement in plant defense mechanisms.

## 2. Small RNA Biogenesis and Mode of Action

**Figure 2:**
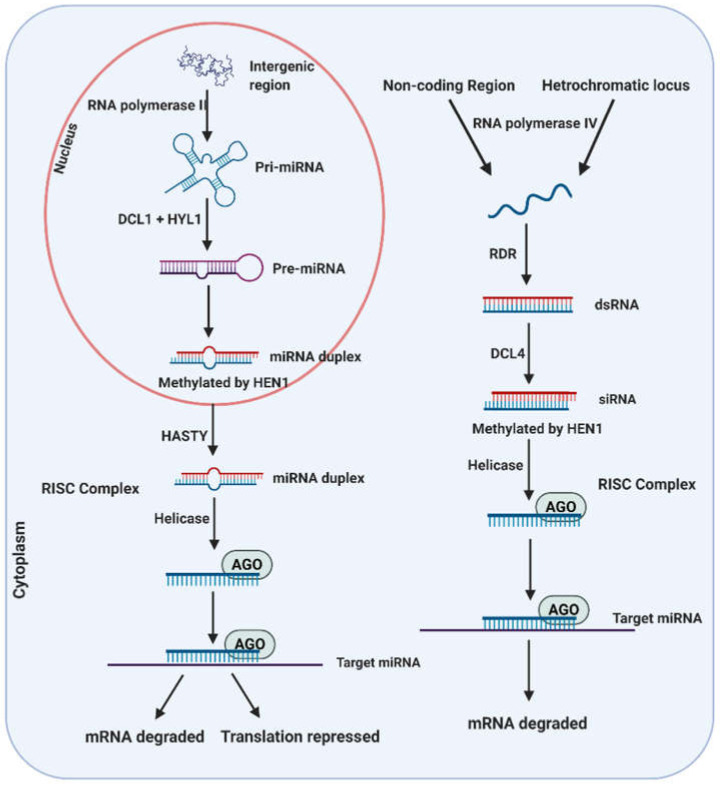
Schematic representation of biogenesis of sRNA (miRNA and siRNA) in plants. The miRNAs are processed from intergenic genome regions. Plant microRNAs are transcribed by RNA polymerase II and Dicer-like 1 (DCL1) in the presence of the protein Hyponastic Leaves 1 (HYL1) from the transcripts located in intergenic regions form miRNA duplexes. The miRNA duplexes are methylated by the Hua Enhancer 1 (HEN1) and HST protein (HASTY) which moves duplex miRNA from the nucleus to the cytoplasm. The guide miRNA strand binds AGO, making it accessible to the RNA-induced silencing complex (RISC) to carry out the translation repression or mRNA degradation. The biogenesis of siRNA depends on the type (Ra-siRNA heterochromatic locus and Ta-siRNA non-coding regions) of siRNA being synthesized. Single-stranded precursors are processed for the respective siRNAs by the activation of RNA pol IV or through miRNA-mediated cleavage. dsRNA is transcribed with the help of RNA-dependent RNA polymerases (RdRps) from the ssRNA precursor. DCL4 cuts dsRNAs to produce a duplex of siRNA that is methylated through HEN1. A helicase untwines the siRNA duplex and binds to RISC and then it binds to its target mRNA siRNA and degrades the sequence.

miRNAs are single-stranded endogenous RNAs with a regulatory function of gene expression at the post-transcriptional level, either through the degradation of target messenger RNAs or by translation inhibition [36]. The biogenesis of a miRNA starts with the transcription of a nuclear gene (MIR gene) by RNA polymerase II. The promoter region of MIR genes contains transcription initiation boxes (TATA type) and regulatory elements, having the characteristic of binding to various types of transcription factors (W, MYC, MYB boxes, among others) [37]. Transcription of the MIR genes produces a primary miRNA (pri-miRNA) that is stabilized by the addition of a methylguanosine (5 ’end), and a polyadenylated tail (polyA, 3′ end). The pri-miRNA transcripts adopt a hairpin-shaped structure that is processed in two sequential stages by a Dicer-like1 ribonuclease (DCL1), which generates an intermediate precursor (pre-miRNA) and is subsequently processed to form a miRNA/miRNA* duplex, also known as miRNA-5p/miRNA-3p duplex (Figure 2) [38]. Although miRNA biogenesis involves a main Dicer-like protein, DCL1, a recent report has suggested that DCL3 and DCL4 can also contribute to the processing of miRNA precursor transcripts [39]. The activity of DCL3 or DCL4 has been seen in longer miRNAs (23–25 nucleotides) from an evolutionary point of view.

In most cases, only one of the miRNA duplex chains is functional, while the other strand is ejected and degraded. However, miRNAs have also been described in which the two duplex chains are functional, such as miR393 [40]. The miRNA/miRNA* duplex is methylated through a HUA ENHANCER 1 (HEN1) enzyme and transported to the cytoplasm by HST (HASTY) proteins. Once in the cytoplasm, the functional miRNA chain is incorporated into the RNA-induced silencing complex (RISC), which is processed and loaded into Argonaute (Ago) proteins. RISC guides the miRNA towards the target transcript (sequence complementarity recognition) and is responsible for silencing the target gene. Other RNA-binding proteins necessary for the biogenesis process of miRNAs are HYPONASTIC LEAVES 1 (HYL1), NEGATIVE ON TATALESS 2 (NOT2), DAWDLE (DDL), TOUGH (TGH), and SERRATE (SE) [41]. In recent years, massive sequencing techniques have been used to identify the population of miRNAs in many plant species [42,43]. The highest numbers of miRNA sequences deposited in miRBase include those for rice (713) and *Arabidopsis* (427) [42]. However, there are very few plant miRNAs for which information is available on their function and the physiological processes in which they participate.

The class of siRNAs includes different types of small RNAs, such as natural antisense siRNAs (nat-siRNAs), heterochromatic siRNAs (hc-siRNAs), phased secondary siRNAs (pha-siRNAs), and trans-acting siRNAs (ta-siRNAs) [34]. The difference between miRNAs and siRNAs resides in the type of molecule from which they originate (single-stranded or double-stranded RNA), and also the biogenesis and functional mechanism. Unlike miRNAs that are generated from precursors of single-stranded RNAs, the endogenous siRNAs come from a double-stranded RNA that is caused by the activity of an RNA-dependent RNA polymerase (RdRp) [44]. The production of each type of siRNA from the corresponding precursor requires the participation of specific members of the RDR family of proteins (RDR2, RDR6), and DCL proteins (DCL2, DCL3, or DCL4). For example, hc-siRNAs require RDR2 and DCL3, while the production of ta-siRNAs requires RDR6 and DCL4 [45]. The biogenesis of phasiRNAs is similar to that of tasiRNAs. The prefix “phasi” refers only to the “phased” feature, and phasiRNA is different from tasiRNA, which has been experimentally proven in transcription [46]. RDR6, DCL1, and DCL2 are involved in the production of nat-siRNAs [44]. Thus, an essential difference between miRNAs and siRNAs is the dependence on RDR for the production of siRNAs, but not for the production of miRNAs. The siRNAs are incorporated into RISC to perform their function [47]. A specificity is observed in the role of members of the AGO family and the different types of small RNAs. While AGO1 preferentially recognizes miRNAs, AGO4 recognizes hc-siRNAs [48]. The hc-siRNAs (24 nucleotides) are the most abundant siRNAs in plants and participate in transcriptional gene silencing through RNA-directed DNA methylation.

### Function of Small RNAs in PTI

**Figure 3:**
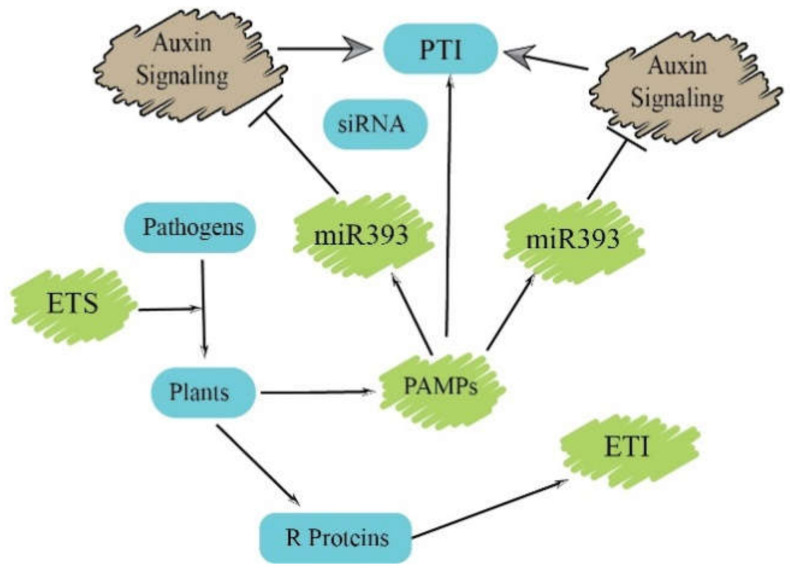
PAMP-triggered immunity (PTI) and effector-triggered immunity (ETI) regulated by small RNAs.

In the beginning, it was demonstrated in *Arabidopsis* that miRNAs are involved in PTI responses. The treatment of flagellin22, a 22 amino acid peptide derived from the general elicitor flagellin, leads to the increased accumulation of miR393, which in return represses the expression of genes coding for auxin signaling receptors such as AFB genes. The miR393a-overexpressing plants suppress auxin signaling and confer resistance against the bacterial pathogen Pseudomonas syringae pv. tomato (Pto) DC3000, whereas plants constitutively expressing *AFB1* showed rapid growth of Pto DC3000 bacteria [40]. These studies establish a clear molecular connection between miR393 and auxin signaling in PTI responses, suggesting that, directly or indirectly through hormonal regulation, miRNAs can regulate plant defenses in a precise manner by responding to pathogen infection (Figure 3). miRNAs have also been implicated in the regulation of other hormone signaling pathways, for example, the ABA signaling pathway is regulated by miR159 through its target *MYB101* and *MYB33* transcripts, whereas miR319 targets the *TCP* transcription factor involved in regulating the JA biosynthesis pathway [49].

Studies have shown that miR393 is involved in regulating three different pathways to resist pathogen infection. First, miR393 regulates SA-mediated signaling, which primarily induces a defense response against biotrophic infection [50]. One of the critical components in the SA signaling pathway is non-expressor of pathogen-related genes 1 (NPR1), capable of interacting with transcriptional factors (TGA type) that recognizes the promotors of defense genes [51]. Secondly, miR393*, derived from the MIR393b transcripts, modulates the exocytosis of the SA-induced antimicrobial protein PR1 [52]. Thirdly, miR393 redirects the secondary metabolic flow to promote disease resistance. *ARF1* and *ARF9* are two transcription factors that promote the biogenesis of camalexin, an indolic phytoalexin that is most effective against necrotrophic fungi such as *Alternaria brassicicola* [53,54]. Besides this, a large number of sRNA pathway proteins also help in initiating the defense against pathogen attack. These are (a) Dicer or Dicer-like endoribonuclease (DCL), involved in the generation of sRNAs; (b) AGOs which induce silencing of the targeted gene with RISC; and (c) RdRps which are engaged in the production of double-stranded sRNA precursors. These proteins are thought to be linked with ETI and PTI during fungal and bacterial pathogen attack [55,56]. Several studies have shown the role of DCL protein in the mechanism of defense against different pathogens. In *Arabidopsis*, *dcl1-9* [40] and *dcl1-7* [57] mutants were more susceptible to fungal and bacterial infections. Certain proteins, such as DCL-4 and AGOs, also play a significant role in preventing the invasion of viruses, bacteria, and fungi.

miR393 suppresses TIR1/AFB2-mediated auxin signaling which prevents the suppression of SA, and subsequently confers enhanced resistance against *P. syringae* bacteria in *Arabidopsis* [40]. Furthermore, miR393-mediated regulation of auxin signaling is also observed in rice (*Oryza sativa*). miR393 regulates the expression of auxin receptor gene homologs (*OsTIR1* and *OsAFB2*) in rice, and miR393 overexpression results in early flowering, hyposensitivity to auxin, and reduced tolerance toward drought and salt stress [58]. Thus, miR393 appears to have a clear function in hormonal signaling pathways related to the defense response. In addition to miR393, other bacterium-regulated miRNAs, such as miR160 and miR167, also play essential roles in plant defense [59,60]. In rice, the overexpression of miR160a results in a higher accumulation of H_2_O_2_ at the infection site and an induction of defense gene expression against blast fungus disease [61]. The expression of miR167 is shown to be regulated during *Agrobacterium tumefaciens* infection, a bacterium used for inserting genes into the plant genome. An oncogenic strain of *Agrobacterium tumefaciens* induces miR167 expression at the infiltration regions, while a strain lacking tumorigenic properties did not induce the expression of miR167 [62].

Recent studies showed that miR160a is a positive regulator PTI, while miR398 is a negative regulator of PTI and modulates the deposition of callose in resistance against *P. syringae* [60]. Similarly, in *Arabidopsis*, the accumulation of miR398 is altered during bacterial infection, and miR398 targets and regulates the expression of two of three Cu/Zn- superoxide dismutase (SOD) transcripts (*CSD1* and *CSD2*). SOD proteins are metalloenzymes that detoxify ROS and protect cells from the oxidative stress associated with pathogen infection [63]. In *Arabidopsis*, upon infection by avirulent strains of Pst DC3000 (*avrRpm1* or *avrRpt2*), miR398 decreased in abundance, which was associated with an increase in the transcript level of *CSD1* and *CSD2* and alleviated oxidative stress levels. In rice, the overexpression of miR398b results in a higher accumulation of H_2_O_2_ at the infection site and the induction of defense gene expression, such as the activation of *PR1* and *PR10* genes [61].

In *Arabidopsis*, during *P. syringae* infection, miR390 expression declines with a subsequent increase in the expression level of *TAS3*, activating the production of ta-siRNAs that direct the regulation of *ARF3* and *ARF4* genes required in auxin signaling [64]. The overexpression of miR400 or miR844 in *Arabidopsis* led to increased susceptibility to *P.syringae* pv. tomato DC3000 and to the fungus *Botrytis cinerea* [65]. A recent study showed that miR160a positively regulates the accumulation of callose. At the same time, miR398 and miR773 are negative regulators of PAMP-induced callose deposition, and by modulating the deposition of callose, these miRNAs provide disease resistance against *P. syringae* in *Arabidopsis* [60]. *Blumeria graminis*, a powdery mildew fungus, triggers the production of many miRNAs in wheat (*T. aestivum*) and, among these, miR167, miR171, miR408, miR444, and miR1138 are involved in PTI [66].

## 3. The Role of Small RNAs in ETI

To generate more durable disease resistance against pathogen effectors, plants have evolved an R gene-mediated defense mechanism, also referred to as ETI. The recognition of pathogen effectors by R proteins triggers robust cellular changes through generating an HR at the infection site. R proteins have conserved domains, and the most common group of R proteins has a nucleotide-binding site (NBS) located in the central region and a leucine-rich repeat domain positioned at its C-terminal end [67]. Another type of R protein has only an extracellular leucine-rich repeats (LRR) domain, such as the proteins of the Cf family of tomato (*Solanum Lycopersicum*) that confer resistance to the fungus *Cladosporium fulvum* [68]. The majority of disease resistance genes in plants encode NBS-LRR proteins to allow the recognition of pathogens [69]. The activity and quantity of R proteins under normal conditions are sustained at a lower level to save resources for plant growth and development. However, when plants come under attack, PTI can be suppressed by the pathogen effectors, which trigger ETI through the subsequent upregulation of R genes. Plants with over-activated NBS-LRR proteins or constitutive exposure to PAMPs exhibit stunted growth and development, which is due to the tradeoff between growth and defense [70]. Hence, the activity of plant defense response is tightly coordinated, and in an infection situation, plant growth can be compromised due to plant defense. A recent study in apple demonstrated that sRNAs indirectly regulate the expression of the R gene through targeting the genes related to the co-expression of R genes and hence contribute to negative feedback loops [71]. In *Arabidopsis*, recognition of peronosporaparasitica 5 (*RPP5*) locus R genes were downregulated in plants overexpressing SUPPRESSOR OF NPR1-1, CONSTITUTIVE 1 (SNC1), and the overexpression of SNC1 triggers the downregulation of RPP5. For instance, mutants defective in sRNA biogenesis, such as *dcl4-1* and *ago1-36*, were shown to have elevated levels of SNC1 transcripts, suggesting that during pathogen infection, SNC1 is repressed through an sRNA pathway, which most likely enables the expression of R genes [72].

Recent advances in plant biology have led to the identification of new miRNAs that target NBS-LRR genes in several plant species, including sugarcane [73], grapevine (*Vitis vinifera* L.) [74], and Citrus trifoliata [75]. In several cases, it has been experimentally proven that miRNAs mediated the regulation of NBS-LRR gene functions in enhancing plant defense. Md-miRLn11 targets the NBS-LRR-encoding gene in *Malus domestica* [76]. In 40 apple varieties subjected to the bacterial pathogen *Alternaria alternata* f.sp. *mali*, *Md-NBS* showed higher expression levels in resistant varieties compared to the susceptible varieties. Transient expression of *Md-miRLn11* and *Md-NBS* in leaves also supports the conclusion that *Md-NBS* mediates resistance against apple leaf spot disease and that miRLn11 represses *Md-NBS* [76]. In tobacco (*Nicotiana. benthamiana*), two miRNAs, miR6019 and miR6020, regulate TIRNBS-LRR (TNL)-type receptor genes, and the cleavage caused by these miRNAs further triggers the production of 21-nt phasiRNAs, which reinforce the suppression of R genes. The overproduction of miR6019 and miR6020 weakens TIR-NBS-LRR protein N-mediated resistance against tobacco mosaic virus [77]. The superfamilies of miRNAs miR482 and miR2118 regulate R genes of the NBS-LRR type in tomato [78]. The bioinformatics-based analysis suggested that miR482 targets 58 of the 168 R genes, with a priority for coiled-coil (CC)-NBS-LRR (CNL) mRNAs [78]. Intriguingly, miR482 is predicted to bind in the *P*-*loop region* of the transcript, a highly conserved signature structure of NBS-LRR proteins. This allows miR482 to regulate a larger group of NBS-LRR genes, and such actions of miR482 can trigger the generation of phasiRNAs, which leads to a simultaneous silencing of *R* genes [79]. In tomato, miR482 and miR5300 are induced upon fungal *Fusarium oxysporum* treatment, and these two miRNAs target the three NB domain genes and *tm-2*, a susceptible allele of the *Tomato Mosaic Virus* resistance gene, and when treated with *F. oxysporum*, these genes demonstrated higher expression levels in the resistant cultivars compared to susceptible varieties, suggesting that miR482/miR5300-mediated regulation of *NB* genes in tomato plays a crucial role in resistance against fungi [80]. miR472 was shown to be involved in regulating the PTI and ETI responses in *Arabidopsis* through post-transcriptional control of NBS-LRR (CC-NBS-LRR) genes [81]. Besides, the miR9863 family triggers the generation of 21 nt long phasiRNAs, which in concert with miR9863 form a regulatory network for repressing the expression of group 1 *Mla* alleles, which encode CC-NBS-LRR receptors. *miR9863* regulates distinct *Mla alleles* in barley, and the overexpression of miR9863 reduces MLA1-triggered resistance against powdery mildew fungus [82].

**Figure 4:**
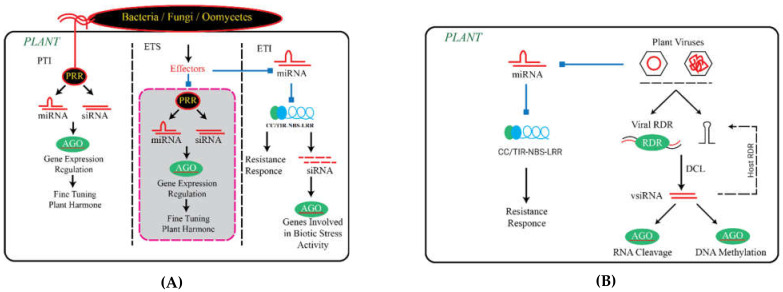
The role of sRNAs in plant immunity. (**A**) The role of small RNAs (sRNAs) during the interaction between plant and pathogens. (**B**) Plants defend against virus attacks by silencing the viral DNA/RNA genome through RNAi. Both microRNAs (miRNAs) and virus-derived small interfering RNAs (vsiRNAs) are involved. CC, coiled-coil; PTI, pathogen-associated molecular pattern-triggered immunity; ETS, effector-triggered susceptibility; ETI, effector-triggered immunity; PRR, pattern recognition receptor; DCL, dicer-like protein; HEN 1, HUA ENHANCER 1; LRR, leucine-rich repeat; NBS, nucleotide-binding site; RDR, RNA-dependent RNA polymerase. CC, coiled-coil; TIR, transport inhibitor response; LRR, leucine-rich repeat; RDR, RNA-dependent RNA polymerase.

In addition to miRNAs, other sRNAs also participate in regulating the components of ETI (Figure 4). The first plant endogenous siRNA (nat-siRNAATGB2) was found in *Arabidopsis*. During infection, this sRNA acts as a positive regulator of ETI triggered by avrRpt2 [83]. The 22 nt long nat-siRNAATGB2 is generated from the overlapping region of the antisense gene pentatricopeptide repeats-like (*PPRL*). Nat-siRNAATGB2 silences *PPRL* and inhibits the negative stimulatory effect of *PPRL* on the RPS2 signaling pathway. The *PPRL overexpressing* lines show *delayed* HR in response to *Pst* (*avrRpt2*), and it also facilitates the growth of *Pst* (*avrRpt2*). However, such a response of *PPRL* is not observed in *Pst* (*avrRpm1*) [83]. These findings suggest that *PPRL* is a negative regulator of RPS2-dependent ETI against *Pst avrRpt2*. Another siRNA induced by the effector avrRpt2 is AtlsiRNA-1, a 30 to 40 nt long sRNA, which is generated from the SRRLK or AtRAP natural antisense transcript pair [84]. AtlsiRNA-1 represses the expression of *AtRAP* mRNA, which is most likely due to decapping and XRN4-mediated degradation. RAP domain-containing protein is encoded by *AtRAP*, which plays an important role in disease resistance. *AtRAP* silencing results in improved disease resistance against both avirulent *Pst* (*avrRpt2*) and virulent *Pst* (EV) [84]. miR863-3p is highly induced by *Pst* (*avrRpt2*) infection and sequentially silences negative and positive regulators of plant immunity through two different modes of action. MIR863-3p first suppresses typical receptor-like pseudokinase1 (ARLPK1) and ARLPK2 through mRNA degradation to improve defense responses quickly after infection. ARLPK1 interacts with AKIK1, forming a negative feedback loop to attenuate immune responses after successful defense. Then, in the later stages of infection, miR863-3p lowers SERRATE (SE) by translational inhibition to attenuate defense signals by reducing the level of miR863-3p, which depends on SE accumulation, thus forming a negative feedback loop to attenuate plant immunity [85].

Previously, the complementary strand of the miRNA (miRNA* (star) strand), was considered as an unwanted byproduct that is eventually degraded. However, recent research has challenged this view by demonstrating the distinct role of miR393* in regulating the defense response of *Arabidopsis*. miR393* was shown to regulate the expression of the *MEMB12* gene that encodes a SNARE protein of the Golgi apparatus, which is involved in vesicular transport and modulates the exocytosis of antimicrobial pathogenesis-related PR1 protein [40,52]. Plants overexpressing miR393b* showed phenotypes similar to those of the *memb12* mutant with increased exocytosis of PR1, which subsequently enhances plant resistance upon avrRpt2 infection. miR393 targets *AFB1* but miR393b* does not. The overexpression of *AFB1* does not affect the growth of *Pst* (*avrRpt2*) [40]. Thus, two sRNAs, miR393 and miR393b, produced from the same duplex, have different functions in facilitating PTI and ETI.

## 4. miRNAs Involved in Environmental Stress Responses

Plants have developed complex mechanisms to overcome various environmental stresses. miRNAs have very versatile functions and appear to participate in response to biotic and abiotic stresses. Environmental stress causes an increase or decrease in the expression of specific miRNAs, and plants have also developed mechanisms for the synthesis of new miRNAs to protect themselves against different stress factors. The role of miRNAs in plant development is well established, while less is known about their role in environmental stress response.

In the recent past, miRNAs regulated by various stress conditions were identified in plants, which include lack of nutrients [86], drought [87], low temperatures [88], salinity [89], and pathogen infection [90], suggesting that miRNAs are regulated in various ways in response to environmental stress. Jones-Rhoades and Bartel revealed that, in *Arabidopsis*, miR395 levels are increased in sulfate starvation conditions [91], which suggests that miRNAs are not only synthesized through developmental processes but also due to environmental factors. miR395 targets the ATP sulfurylase APS4 enzyme, which catalyzes the assimilation of inorganic sulfates and accumulates in low-sulfur conditions [92]. A study conducted in *Arabidopsis* showed that a quarter of expressed sequence tag (EST)-containing miRNAs are induced by stress conditions, which shows the potential role of miRNAs in plants against different stress responses [93]. Sunkar and Zhu constructed a small RNA library and identified several new miRNAs in *Arabidopsis* exposed to various stress conditions, which include high salinity, dehydration, ABA, and cold ambient temperature [94]. miR393 and miR319c showed increased expression levels during exposure to different stress factors; however, the expression of miR389a, miR397b, and miR402 declined under stress treatments including cold, dehydration, salinity, and ABA [94]. The transcriptome analysis of *Populus* revealed that most of miRNA targets are associated with development and stress/defense. These miRNAs are induced during environmental stress conditions and participate in the protection system of plants for structural and mechanical fitness [95]. GA and ABA play a critical role in response to various environmental stresses. These two phytohormones regulate the expression of miRl59 and subsequently control the development of flower organs [96,97]. An experiment conducted on the mutants of some miRNA families demonstrated hypersensitivity to osmotic stress and ABA [89]. The modification of miRNA targets and induced changes in mutated genotypes showed better responses against abiotic stresses compared to wild-type plants [98]. Recently, a study investigating the impact of abiotic stress treatment on the stress-tolerant *7B-1* tomato mutant and wild-type plants showed that several miRNAs were differentially expressed under various stress conditions. miRNA159 was significantly downregulated under cold, ABA, and NaCl treatments. miRNA166 showed an altered expression pattern in root and hypocotyl tissues. Similarly, miR159, miR472, and miR482 were also inversely expressed in the hypocotyl compared with roots [99]. In another study, expression patterns of different miRNAs were analyzed in resistant and susceptible soybean cultivars against drought stress and Asian soybean rust (*Phakopsora pachyrhizi*). Some miRNAs were differentially expressed in response to drought stress and rust infection. Of these, MIR-Seq11 targets the peroxidase precursor mRNAs and was differentially expressed between the mock and rust-susceptible genotype (Embrapa 48). In contrast, the soybean rust-resistant genotype (PI561356) did not show any change in expression pattern. Furthermore, MIR-Seq11 expression was upregulated in roots of a drought-susceptible genotype (BR 16) compared to the controls, while it responded similarly in treated and untreated drought-tolerant cultivars [100].

In addition to miRNAs, other types of sRNAs also regulate the expression of target genes to overcome stress response in plants. In *Arabidopsis*, a nat-siRNA derived from SRO5 and P5CDH plays a crucial role in the management of salt-induced oxidative stress. The induction of *SRO5* by salt stress complements P5CDH for the production of double-stranded RNA (nat-siRNA) processed by an siRNA biogenesis pathway. The 24 nt nat-siRNA downregulates the expression of *P5CDH* by causing mRNA cleavage. The onset downregulation of the P5CDH transcript activates DCL1, which produces a more specific 21 nt long siRNA (P5CDH nat-siRNAs). These nat-siRNAs direct the cleavage of P5CDH mRNAs to suppress proline degradation and enable the accumulation of proline [101]. P5CDH downregulation contributes to P5C-mediated ROS accumulation [101] and induces SRO5 involved in ROS detoxification [101]. This entire system is mediated by siRNAs irrespective of transcription factors or signal transduction factors and allows the plants to respond quickly to salt and oxidative stress.

## 5. miRNAs and Secondary Metabolites in the Defense Response of Plants

Plants produce a large number of very diverse secondary metabolites, which can potentially participate in the defense against pathogens. Probably the most studied example of metabolite production is the phytoalexins. In *Arabidopsis,* the most abundant indole phytoalexin is camalexin, which is synthesized from tryptophan [102]. Several miRNAs have been shown to affect the metabolic flow of camalexin and other tryptophan derivatives that, in general terms, are known as indole glucosinolates and contribute to the defense response of plants in different pathosystems [103,104]. Transcriptomic analysis of Swertia revealed several miRNAs associated with the synthesis of secondary metabolites, including miR-156a, miR-166a, miR-168, and miR-11320, which target metabolic enzymes, such as acetyl-CoA acetyltransferase, aspartate aminotransferase, premnaspirodiene oxygenase, and phosphoglycerate mutase. miR393 was shown to promote the defense response through redirecting secondary metabolic flow [105]. miRNAs regulate the production of defensive metabolites through a change in gene expression in elicited or infected plants. Light-stimulated miRNAs in *Solanum tuberosum* L. are essential regulators of lipid biosynthesis, alkaloid metabolism, and cellulose catabolism [106]. Stress with cadmium in *Brassica napus* L. uncovered miRNAs implicated in the regulation of the biotic stress response, transcription factors and secondary metabolite synthesis [107]. Transcription factors such as *ARF1* and *ARF9* promote camalexin biogenesis, which has an effective response against *Alternaria brassicicola*, a necrotrophic fungus [54], and also represses glucosinolate accumulation, which exerts a toxic effect on a wide range of bacteria [108].

Flavonoids represent another critical group of secondary metabolites in plants and are synthesized from the amino acid phenylalanine, through the phenylpropanoid metabolic pathway. Plants produce three main groups of flavonoids: flavonols, anthocyanins, and proanthocyanins. These compounds have multiple biological functions, including anti-inflammatory, antioxidant, and anticarcinogenic properties, and are synthesized through interaction with the environment, microorganisms, and other plants [109]. Phenylalanine ammonia-lyase (PAL) initiates phenylpropanoid catabolism by catalyzing the production of cinnamic acid, which is consequently converted to p-coumaric acid and 4-coumaroil-CoA by the activity of the enzymes cinnamate-4-hydroxylase (C4H) and 4-coumate coenzyme A ligase (4CL). Phenylpropanoid 4-coumaroil-CoA is the precursor for the synthesis of flavonols and anthocyanins [110]. Flavonoid biosynthesis is highly regulated by miRNAs [111,112,113], in which interactions of different families of transcription factors that participate in the various branches of this route are observed in a modular way, which include members of the R2R3 family of MYB transcription factors (*AtMYB11*, *AtMYB12*, and *AtMYB111*), basic helix-loop-helix (bHLH), and WD40 [114]. In *Arabidopsis*, miR858a regulates the activity of several MYB transcription factors that play a role in the flavonoid biosynthesis pathway. miR858 overexpression negatively regulates MYB transcription factors, and the interference with the miR858 target mimic (*MIM858* lines) induce a higher expression of MYBs, which redirects the metabolic flux toward flavonoid synthesis [115]. Similarly, plants with a higher level of miR*858* are more susceptible to fungal infection, whereas *MIM858* lines show resistance against pathogens [115]. A study in *Withania somnifera* revealed that miR477 and miR530 in leaf and miR159 and miR5140 in root tissue were involved in regulating the synthesis of secondary metabolites [116]. Another study showed that miR8154 and miR5298b increase the synthesis of flavonoids and phenylpropanoids in subcultured Taxus cells [117].

Although the mechanism by which miRNAs regulate secondary metabolites to participate in plant defense against pathogen attack has not been characterized thoroughly, it is based on the ability of these metabolites to act as antioxidants or chelating agents. The role of miRNAs in regulating secondary metabolism is a relatively new field of study. Understanding and knowledge related to the rational regulation of secondary metabolites would help to formulate novel strategies to strengthen the plant defense system.

## 6. Role of miRNAs in Defense Priming

The priming phenomenon in plant defenses has been described for more than ten years. The mechanism of defense priming induces a physiological state in which the plant is programmed to trigger the activation of defense responses in a faster, more intense, and long-lasting period of time against pathogen attack and stress response [118]. The priming or defense enhancer mechanism has been developed by plants as an adaptive feature for the adjustment of defense responses under unpredictable environmental conditions. Priming events may be triggered as a result of interindividual or interspecies communication, such as treatment with specific compounds (BABA, bacterial oligosaccharides) and interaction with beneficial microorganisms (e.g., mycorrhizal fungi, rhizobacteria), as well as metabolic alterations to acquired systemic resistance. In a recent study, it has been observed that defense priming can pass from one generation to another, indicating the implication of priming as an epigenetic component of transgenerational defense [119]. Molecular studies about the phenomenon of defense priming have been associated with changes in chromatin and the accumulation of mRNA genes regulated by miRNAs with a role in defense signaling, such as transcriptional regulators, protein kinases, and pattern recognition receptors [120]. In an infection situation, miRNAs regulate the genes involved in the defense priming process, thus allowing plants to respond more quickly and intensely by mounting a more robust defense response to counteract the pathogen infection [121]. Reduced activity of miR396 confers broad-spectrum resistance against hemibiotrophic and necrotrophic fungal pathogens. During the fungal infection, levels of miR396 decreased gradually, therefore allowing its transcription factor target gene *GROWTH-REGULATING FACTOR* (*GRF*) to trigger host reprogramming. miR396-overexpressing plants showed disease resistance due to the super-activation of defense responses and this is in agreement with a priming event. *P. cucumerina* treatment in *MIM396* plants displayed enhanced defense responses through increased H_2_O_2_ accumulation and callose deposition and was followed by transcriptional reprogramming, which leads to an improved immune system against pathogen infection [121]. These findings suggest that miR396 is a crucial player in regulating plant immunity and processes that sustain defense priming. A recent study in *Lotus japonicus* revealed that miR172a is involved in priming cells for infection and functions in the process of infection and differentiation into sink tissues [122]. However, the current information available on the molecular mechanism of miRNAs underlying priming events and their significance in plant resistance is quite limited.

## 7. Perspectives and Biotechnological Applications of miRNAs

Biotechnology applied to the protection of plants against diseases represents a useful strategy for the genetic improvement of plants, complementary to the more traditional cross-breeding techniques. In most species of agronomic interest, there are substantial losses due to attack by pathogens, whose control currently depends on the use of chemical agents. However, the use of phytosanitary products has negative consequences for the environment, with the possible emergence of resistance in the population of microorganisms in the field. Classical methods of crop improvement based on sexual hybridization are not always possible, so it is necessary to develop new strategies for disease control in plants.

The main biotic stresses that limits plant growth and development are caused by pests and pathogens [123]. In the battle for survival, plants have evolved unique sRNAs to regulate the expression of specific genes to protect themselves against pathogen attacks [124]. Years before the discovery of the RNA silencing concept, Sanford and Johnson proposed the idea of parasite/pathogen-derived resistance (PDR) through transforminga pathogen gene fragment into a plant host [125]. The approach of PDR was widely used to obtain antiviral resistance in plants. PDR has been successfully exploited to confer resistance against viruses to various plants, including the model plants *Arabidopsis* and *N. benthamiana* and other important cereal crops such as wheat, rice, and barley [126,127]. Transgenic papaya resistance against papaya ringspot virus (PSRV) is the most successful case of PDR [128]. Later on, scientists discovered the RNA silencing technique, which helped them to develop transgenic plants that express exogenous RNAi targeting essential genes in insects and pathogens that provide increased resistance to pests and pathogenic diseases.

The use of artificial miRNAs (amiRNAs) and miRNA target mimicry have proven to be useful tools to decipher the function of genes of interest [129]. In an amiRNA gene, the mature miRNA sequence of an miRNA precursor is replaced by a sequence (miRNA) designed to recognize a target gene of interest [130]. The artificial target mimic technology is based on an indigenous regulatory mechanism that negatively regulates the activity of specific miRNAs in plants [97,131]. It was first discovered in the case of the Induced by Phosphate Starvation1 (IPS1) transcript, where *IPS1* induces a motif with a complementarity sequence to the phosphate (Pi) starvation-induced miRNA *miR-399*. In this way, miR399 is regulated by IPS1, preventing the miR399 from performing its function on its target transcript [131]. A recent study demonstrated that transgenic plants expressing amiRNAs are more effective in silencing the same target gene and have improved resistance against insect herbivores, compared to the plants expressing hairpin RNAs (hpRNAs) [132].

The source of the sRNA precursor is crucial for generating effective PDR and is strongly associated with the silencing efficiency [133]. Besides the efficacy of silencing, off-target effects and the persistence of sRNAs are the factors in the selection of sRNA. Determining what PDR constructs do not target and whether they have a negative impact on the host seems essential. Meanwhile, target gene selection is also vital. Promising PDR targets should be genes that are important for the growth and development of pathogens, or perform crucial functions in the plant–pathogen interaction. The moderate stress-induced or tissue-specific overexpression of multiple MIR genes in different plant species has been shown to improve desirable agronomic characteristics and also have the potential of enhancing the productivity and quality of crops. However, pleiotropic phenotypes were also observed in these transgenic events as a consequence of the vast acting network of the miRNAs. The identification and characterization of *cis*-regulatory elements in the MIR gene promoter region will provide a better understanding of the TFs associated with disease resistance and how these MIR genes control plant defenses. From this, the type of promoter to use can be better chosen; in some cases, the canonical promoter sequence can be used. Besides, artificial MIR gene silencing is a powerful strategy that is more precise and reliable compared to other RNAi-based strategies in regulating the targeted mRNA. Nevertheless, its effectiveness also depends on the sequence of promoters chosen to drive its expression. In rice, the overexpression of miR160, miR169, miR398, and miR7695 results in disease-resistance in transgenic rice [134], supporting the potential of miRNA in disease resistance. Overexpression driven by specific promoters is a powerful approach to obtain desirable effects in plants [135]. The use of these biotechnological tools that allow the silencing or activation of miRNAs involved in defense against pathogen attack could be of great utility for obtaining resistance to pathogens in crops [136,137].

The social concern about the use of genetically modified organisms (GMOs) must be taken into account by the scientific community. It is better to use new methodologies that prevent the integration of foreign DNA into the plant genome to obtain disease-resistant transgenic plants. The technology developed for gene editing and directed mutagenesis based on the clustered regularly interspaced short palindromic repeats/Cas9 (CRISPR/Cas9) system can be very useful for modifying the expression of MIR genes or their target genes [138]. The CRISPR/Cas9 non-homologous end-joining (NHEJ) strategy can be used to insert transgene-free *indels* with high specificity or knockout genes in different crops for the improvement of plant disease resistance [139]. In recent years, this technology has been applied to delete genes of interest, including MIR genes [140]. Moreover, it has high editing efficiency, a high percentage of homozygous mutants in the T1 generation, a simple binary vector design, and cloning combined with active nanoparticles. Moreover, the CRISPR/Cas9 system-mediated transcriptional regulation of MIR genes using deactivated nucleases has allowed for the improvement of desired agronomic traits. Although it may be a transgenic-dependent technology with the potential for off-target transcriptional modulation, nuclease expression driven by the tissue-specific or induced promoter, the topical delivery of CRISPR ribonucleoprotein, and *Agrobacterium tumefaciens*-mediated transient delivery can overcome these drawbacks. Unlike for the nucleases of DNA, Cas13a acts with high specificity directly on the RNA molecules. Although there are not many findings in plant systems yet, the latest results obtained in mammalian cells are very encouraging. In this way, it enables the knockdown of pri- and pre-miRNA, mature miRNA, e TMs (endogenous target mimics), circular RNAs (circRNAs), and mRNA, both cytoplasmic and nuclear.

In addition, a transgene-free approach correlated with the possibility of tissue-specific and site-specific RNA editing through the topical delivery of CRISPR ribonucleoprotein or *Agrobacterium tumefaciens*-mediated transient delivery. Another transgene-free approach is based on the topical delivery of linear or structured pre-miRNA and mature miRNA. Although not yet completely developed in plants, its key advantages are a high RNA internalization rate in plant cells, efficient delivery using nanoparticles, few cytotoxic effects, and the possibility of trans-kingdom cross-talking from the topical delivery of structured amiRNAs in plants targeting insect pest or pathogen genes. Furthermore, the potential for the topical delivery of plant miRNAs is associated with improved features in crops (acting as traits or phenotype enhancers). In addition, research on the interkingdom mobility of small RNAs (miRNA or siRNA cross-talk) can provide evidence to enhance the understanding of nematode–, insect–, or pathogen–plant interactions. Additionally, miRNAs could be used as biomarkers for the identification of infection-resistant varieties in populations obtained by traditional cross-linking in genetic improvement programs [98]. Thus, this information enables new biotechnological products to be produced with greater practicality, reduced generation time, and low cost.

### Host-Induced Gene Silencing

Disease control management strategies primarily rely on breeding resistance or tolerance, chemical control, and biological control. The discovery of RNA silencing systems reflects a transgenic approach to the management of diseases. In different crops, host-induced gene silencing (HIGS) using RNA silencing mechanisms and, in particular, silencing the targets of invading pathogens, have been successfully applied in disease prevention. Recent studies have shown that HIGS is a valuable tool in protecting crops from disease in an environmentally friendly manner. HIGS is an RNAi-based mechanism in which small RNAs silence the genes of the pathogens or pests that invade the plant. RNA silencing is a highly conserved mechanism that occurs in most eukaryotes, including plants, fungi, and animals. One of the key features of RNA silencing is the development of small RNAs of 21–30 nucleotides in length that can control gene expression in a sequence-specific manner. The expression of siRNA in transgenic plants provides protection against individual insect species by targeting their genes and other parasitic pests that threaten crops. Transgenic siRNA-expressing plants have proven to be effective in insect control. For example, for the management of cotton insect pests, *Bacillus thuringiensis* (Bt) has been successfully used [141]. Due to its crucial function, ubiquity, and preservation, several studies have reported that heat shock protein 90 (HSP90) is an ideal target [142,143]. The silencing of the chitin synthase gene prevents the insects from hatching [144] and knocking down the segmentation gene, hairy, prevents insect feeding. RNA interference in certain types of nematode species (e.g., root lesion nematodes, root knot nematodes, cyst nematodes, and other ectoparasitic nematodes) has been examined through in planta delivery or soaking, feeding, and injection [145,146]. The knockdown of the pat-10 and unc-87 expression of *Pratylenchus thornei* that targets wheat roots decreases reproduction by 77–81% [147]. Lilley et al., (2007) also identified cysteine proteinase, main sperm protein, C-type lectin, β-1,4-endoglucanase, chitin synthase, FMRF amide-like peptides (flp-14 and flp-18), secreted amphid protein, pectate lyase, chorismate mutase, dual oxidase, splicing factor, SYV46 secreted peptide 16D 10, and secreted peptide as potential RNAi targets [145]. The phenotypic results of these RNA interference experiments suggested a reduced number of nematodes formed or an increase in the male population, indicating that adverse conditions are faced by juveniles [145]. In 2006, the first successful application of HIGS was used to confer resistance against nematodes by shielding the host from infection [148]. A number of nematode factors, including Cpn-1, Y25, Prp-17, tyrosine phosphatase, calreticulin Mi-CRT, parasite gene 8D05, ribosome 3a, 4, synaptobrevin, and spliceosome SR protein, have been shown to be good candidates for HIGS to improve nematode resistance [149]. HIGS against viruses has proved to be a successful technology in the last few years. Using the full-length sequence of the viral replicase (Nib) in transgenic wheat, wheat streak mosaic virus (WSMV) infection is inhibited. Interestingly, transgene mRNA, which did not provide resistance to the virus, can only be identified on one of six lines [150], indicating that post-transcriptional gene silencing is involved in transgenic wheat resistance. More than 70% of crop yield losses worldwide are caused by fungal pathogens, and RNAi strategies have been widely used to characterize gene function and create transgenic lines against fungal pathogens. A novel, revolutionary approach to crop disease control caused by fungi is supported by RNAi-based HIGS. Nowara et al. (2010) revealed that dsRNA targeting the avirulence gene *Avra10*, recognized by the *Mla10* resistance gene, significantly decreased fungal growth in the absence of *Mla10*, and reduced early pathogen development by silencing of 1,3-b-glucanosyltransferases *(BgGTF1* and *BgGTF2)* in wheat and barley [151].

As a new plant genomic modification method, HIGS technology has been used to enhance resistance to various diseases, produce high-quality crops, including the development of seedless plant varieties, improve stress tolerance, and remove fungal toxins [152]. Before RNAi-mediated transgenic plants can be deployed in the field, major challenges need to be addressed. To alter the host defense responses, pathogens and parasites try to deliver small RNAs into the host. In order to minimize invasion by feeding on plants and parasitism, small RNAs may also be introduced into pests and pathogens [153]. Experiments have shown that dsRNA and siRNAs can be effectively transferred into fungal cells. External treatment of siRNA contributes to the downregulation of a particular *Aspergillus* gene, ornithine decarboxylase (ODC), which has been shown to absorb siRNA [154], and the external incubation of dsRNAs and siRNAs in *Botrytis cinerea* significantly inhibits gray mold disease through silencing of the fungal gene *Botrytis DCL1* [155]. However, whether and how dsRNAs and/or siRNAs are transported or even if the parasites’ intact RNAi system is required or not is still shrouded in mist. Recent research suggests that exogenous miRNAs are adequate to regulate the target genes of animals feeding on transgenic plants [156]. These results alter the safety prospects of transgenic plants and should be treated with caution. It is important to identify the methodology of selecting a suitable target and the most appropriate fragment of HIGS to limit or even fully restrict the disease. The success of HIGS is dependent on adequate supplements and the efficient transport of siRNA between the two organisms. HIGS should therefore not be used against necrotrophic fungi because they absorb metabolites and other nutrients from dead host tissues that are unable to provide an adequate supply of siRNAs. In addition, because of functional redundancy, silencing of the individual gene of the pathogens might not be effective to regulate the disease, and the incomplete silencing of mRNA levels may not ensure protein deactivation. The use of the transient silencing method for high-throughput screening will address this downside. Furthermore, in some systems (e.g., soil-borne fungal pathogens and groups of insects that obstruct siRNA uptake) or tissues (e.g., root and fruit), HIGS is not always accessible and in many crop species, genomic editing is not suitable. While recent studies have attempted to express dsRNA in chloroplasts to avoid the drawback of expression in the nucleus, findings demonstrate that when there is random mating in natural conditions, the artificial constructs will be limited to spreading with a lack of chloroplasts [157]. Every organism has its own stable and unique genome for the preservation and transmission of genetic information to future generations. Why does the genomic information stay intact and inheritable for millions of years? A new perspective on this topic is provided by the discovery of RNA silencing. Silencing derived from RNAi can inhibit foreign DNA invasion (viral and transgenic methods). During plant development, a significant immune mechanism of gene expression is the preservation of a low activity of endogenous transposons and repeats, while the regulatory elements and regulations directing the signal pathway have not been identified. The methods used to investigate the pathways of gene silencing are frequently correlated with transgene technology, and future generations need to evaluate and verify them. The HIGS disease management strategy opens new avenues for improving crop yields with deeper insights into the RNAi system.
